# Engineering a PD-L1–sensing synthetic receptor for programmable macrophage response

**DOI:** 10.1186/s13036-026-00708-y

**Published:** 2026-06-06

**Authors:** Ilaria De Martino, Luigi Russo, Matteo Marchetti, Velia Siciliano

**Affiliations:** 1 Istituto Italiano di Tecnologia-IIT, Largo Barsanti e Matteucci, 80125 Naples, Italy; 2 Open University, Milton Keynes, UK; 3 University of Naples Federico II, Naples, Italy

## Abstract

Cellular decision-making relies on the integration of multiple extracellular cues into coordinated functional responses. Synthetic biology provides tools to rewire this process by engineering receptors that convert defined inputs into programmable outputs. Here, we describe a synthetic receptor–based architecture that enables monocytic-like cells to sense an immune-regulatory ligand and conditionally activate a phagocytic program. We engineered a synthetic Notch–based receptor (SNIPR) that detects programmed death-ligand 1 (PD-L1), a broadly expressed immune-regulatory ligand. Upon PD-L1 engagement, the circuit triggers programmable outputs, including expression of a fluorescent reporter or CV1-Fc, as model effector that interferes with CD47-mediated inhibition of phagocytosis. We show that circuit activation scales with PD-L1 levels, partially attenuates PD-1/PD-L1 signaling, and that conditional CV1-Fc expression enhances engulfment of SKOV-3 ovarian cancer cells by THP-1–derived macrophages in vitro. Collectively, this work reframes PD-L1 from an end-point therapeutic target to a programmable input signal for synthetic circuit activation and establishes a modular framework for ligand-responsive control of engineered macrophage behaviour.

**Supplementary Information:**

The online version contains supplementary material available at 10.1186/s13036-026-00708-y.

## Graphical Abstract

**Figure:**
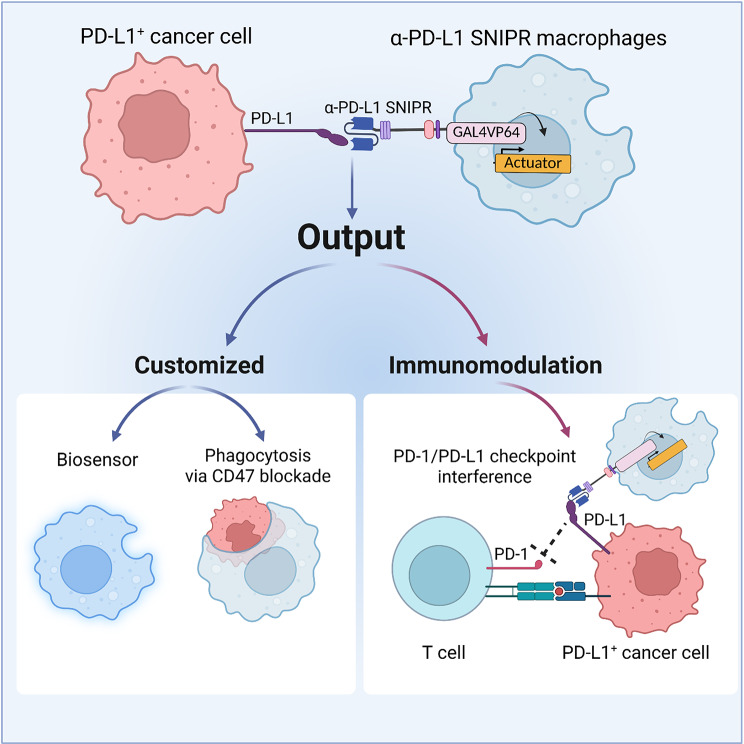

## Introduction

Synthetic biology aims to endow living cells with programmable behaviours by assembling genetic components into modular circuits that sense defined inputs and execute specified responses. In mammalian systems, such circuits can rely on engineered receptors that transduce extracellular molecular signals into intracellular transcriptional programs, enabling cells to function as information-processing units. In this context, synthetic receptors like Synthetic Notch provide a generalizable strategy to decouple signal detection from endogenous gene programs and to rewire cellular responses [1, 2].

To date, several applications of synthetic receptor-based sensor-actuator logic have focused on lymphocytes, while comparatively fewer studies have explored their use in innate immune cells [3].

Macrophages represent an attractive cellular chassis for such investigations. They are highly plastic, capable of integrating diverse environmental signals to shape the immune response in complex scenarios such as solid tumors [4]. Rather than viewing macrophages solely as therapeutic effectors, they can be considered as dynamic systems whose behaviour emerges from the integration of multiple activating and inhibitory cues.

Indeed, in tumor-associated settings, macrophages are exposed to dominant inhibitory signals that constrain phagocytic activity and bias their functional state, skewing them from a pro-inflammatory (M1-like) towards an anti-inflammatory and immunosuppressive (M2-like) state [5–9]. These features make macrophages a compelling model for investigating how synthetic circuits can be used to override or reinterpret endogenous regulatory signals.

Recent studies have begun to explore macrophage engineering using synthetic receptors, including CAR-based designs that enable antigen-directed engulfment [10, 11] and chimeric cytokine receptors (ChCR) that convert immunosuppressive cues intro pro-inflammatory signalling [12]. While these efforts suggest that macrophages can be equipped with engineered signal-processing modules, the above-mentioned works rely on activation of endogenous gene programs. Therefore, general frameworks for integrating extracellular sensing with orthogonal programmable outputs in these cells have thus far remained largely unexplored.

Here, we present a synthetic receptor-based circuit that links recognition of a defined extracellular ligand to controlled execution of a phagocytic program in monocytic-like cells.

We focused on programmed death-ligand 1 (PD-L1) as a model input signal, not as a therapeutic target per se, but as a prototypical immune-regulatory ligand whose expression conveys contextual information about the surrounding cellular environment.

To this end, we engineered a PD-L1-sensing synthetic Notch-based receptor (α-PD-L1 SNIPR), that converts ligand engagement into transcriptional activation of customizable outputs. In THP-1–derived macrophages co-cultured with SKOV-3 ovarian cancer cells, the α-PD-L1 SNIPR triggers the expression of an inducible reporter gene in response to the ligand (sensor output), and of a fusion protein that blocks the CD47 “don’t eat me” signal to enhance phagocytosis in vitro (actuator output).Furthermore, by controlling α-PD-L1 SNIPR expression with endogenous macrophage-specific transcriptional regulatory elements, we introduce an additional layer of context dependence that restricts circuit activation to polarized macrophage.

Together, this work establishes a proof-of-concept framework for programming macrophages behaviour using sensor-actuator circuits that integrate extracellular sensing, decision-making, and effector execution. By focusing on ligand-responsive control architectures in innate immune cells, this work provides design principles that expand the synthetic biology toolkit for systems-level studies of immune regulation.

## Results

### Engineering and validation of a novel α-PDL1 SNIPR receptor in monocytic-like THP-1 cells

To establish a ligand-responsive control module for programming macrophage behavior, we designed a synthetic receptor that converts extracellular PD-L1 recognition into transcriptional activation of a user-defined output. PD-L1 was selected as a model extracellular input based on three system-level considerations: (i) its abundance across diverse cellular contexts, most notably cancer cells [13, 14] (ii) it can serve as a contact-dependent signal to rewire immune cell responses upon interaction with target cells and (iii) its suitability for competitive engagement by engineered receptors.

We implemented this design using the synthetic Notch (synNotch) and SNIPR receptor architectures, which provide contact-dependent activation, transcriptional decoupling from endogenous signaling pathways, and modular exchange of downstream outputs [15–17]. Specifically, ligand binding to the extracellular domain of the receptor triggers proteolytic cleavage of the transmembrane core, resulting in release of the transcription factor that activates an ortoghonal gene expression program.

**Fig. 1:**
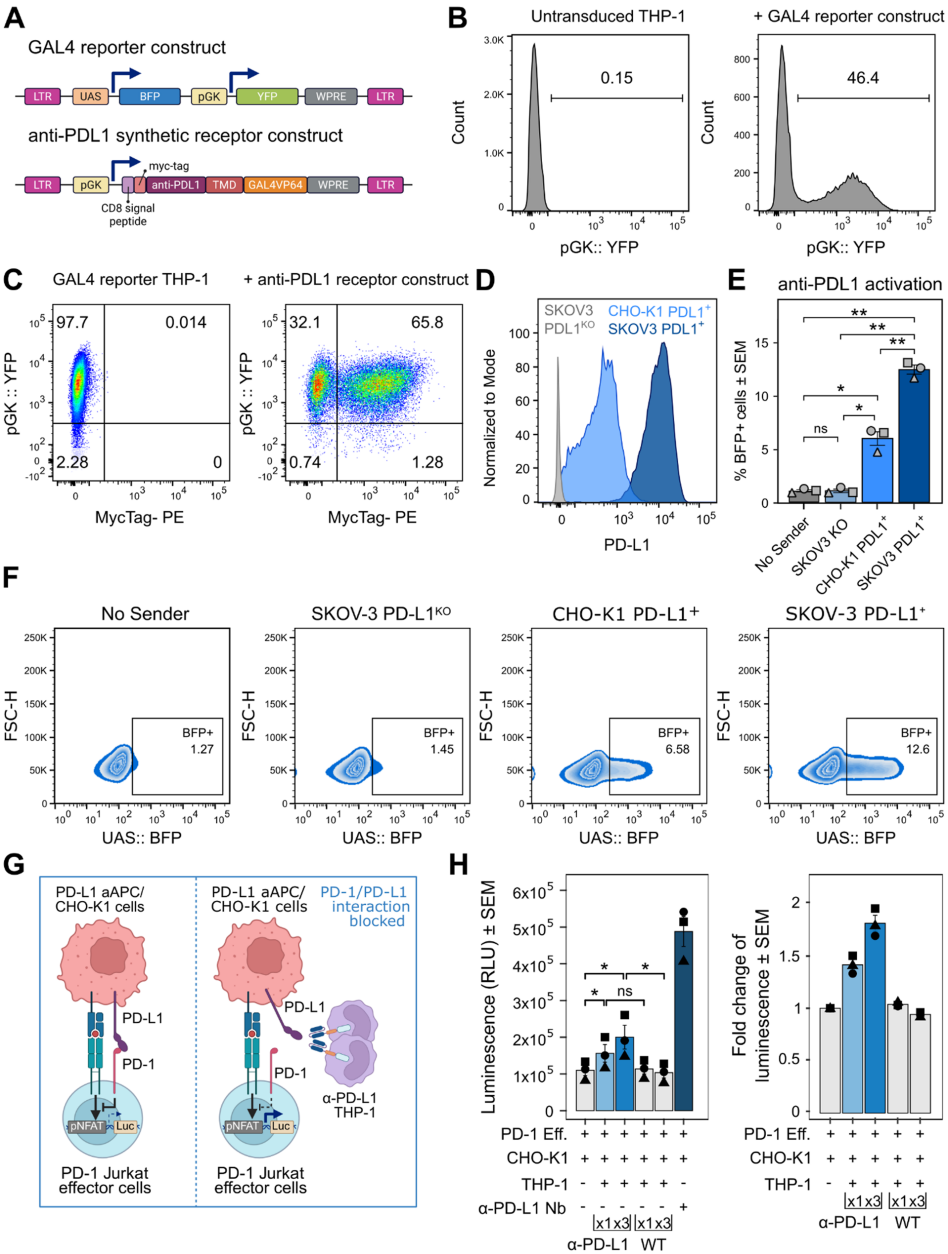
Design and validation of α-PD-L1 SNIPR. (**A**) Schematic of the lentiviral constructs encoding the α-PD-L1 SNIPR and a GAL4-responsive reporter cassette (UAS-BFP). The reporter construct includes a second transcriptional unit driving constitutive YFP expression (UAS-BFP pGK-YFP) for identification of transduced cells. (**B**) Engineering of THP-1 cells with the UAS-BFP pGK-YFP reporter construct. Flow cytometry histograms show untransduced THP-1 cells (left) and YFP expression three days after transduction, prior to cell sorting (right). (**C**) Integration of the α-PD-L1 SNIPR into sorted YFP^+^ THP-1 cells from (**B**). Representative flow cytometry dot plot (right) shows identification of transduced cells by YFP and Myc-tag expression three days post-transduction. (**D**) PD-L1 expression assessed by flow cytometry in PD-L1 knockout SKOV-3 cells (gray), CHO-K1 cells (light blue), and SKOV-3 cells engineered to express homogeneous PD-L1 levels (dark blue). (**E**) Fraction of α-PD-L1 THP-1 cells expressing the BFP reporter when cultured alone or after 48 h of co-culture with the cells from (**D**). Statistical significance was determined using repeated-measures one-way ANOVA with Tukey’s multiple comparisons test (*adjusted *p* < 0.05; **adjusted *p* < 0.01), *n* = 3. (**F**) Representative flow cytometry contour plots corresponding to the conditions shown in (**E**). (**G**) Schematic of the PD-1/PD-L1 signaling interference assay. PD-L1^+^ CHO-K1 antigen-presenting cells (APCs) engage PD-1 on Jurkat T cells engineered with an NFAT-responsive luciferase reporter, resulting in suppressed TCR signaling and low luciferase activity. Binding of α-PD-L1 SNIPR-expressing THP-1 cells to PD-L1 is expected to relieve PD-1–mediated inhibition and restore TCR signaling, leading to increased luciferase activity. (**H**) PD-1/PD-L1 blockade bioassay results shown as absolute luminescence (left) or fold change relative to the basal condition (co-culture of PD-1 Jurkat cells with PD-L1^+^ CHO-K1 cells). α-PD-L1 THP-1 cells were added at THP-1: CHO-K1 ratios of 1:1 or 3:1 (light blue bars). An anti-PD-L1 nanobody served as a positive control for rescue of TCR signaling (dark blue bar). Statistical significance was determined using repeated-measures one-way ANOVA followed by Fisher’s LSD test (*p* < 0.05), *n* = 3

We constructed an α-PD-L1 SNIPR receptor in a lentiviral vector, by fusing an anti-PD-L1 nanobody [18], as putative extracellular domain, upstream the SNIPR core regulatory region [17] and the GAL4 transcription factor as intracellular domain (Fig. 1A). To initially validate PD-L1 sensing, we used as read-out of the receptor activation a blue fluorescent protein (BFP) reporter under the control of a GAL4-responsive promoter (UAS-BFP). This inducible cassette was part of a seperate lentiviral vector together with a second transcription unit encoding a consistutively expressed YFP reporter [17] (Fig. 1A).

The characterization of the receptor activation was carried out in the monocytic-like THP-1 cell line, to mimick in vitro the interaction between macrophages and cancer cells. Originating from the peripheral blood of a patient with acute monocytic leukemia, these cells can be matured into adherent macrophage-like cells with the widely used phorbol 12-myristate-13-acetate (PMA) treatment and are therefore commonly used in preclinical models to test novel, engineered-macrophage-based strategies [19]. We confirmed that PMA-treated THP-1 cells acquired macrophage morphology within 48 h and were polarized toward either a pro-inflammatory (M1-like) or an anti-inflammatory (M2-like) state upon stimulation with lipopolysaccharide (LPS) and interferon-γ (IFN-γ), or interleukin-4 (IL-4) and interleukin-13 (IL-13), respectively (Fig. S1A). Polarization was confirmed by increased expression of CD80 in M1-like macrophages and CD206 in M2-like macrophages (Fig. S1B).

Human monocytic-like THP-1 cells were first transduced with the UAS-BFP/YFP reporter construct and YFP^+^ cells were isolated by flow cytometry cell sorting prior transduction with the lentiviral vector encoding the α-PD-L1 SNIPR (Fig. 1B-C). Inclusion of a myc-tag within the extracellular domain of the receptor enabled the identification of receptor-engineered cells. Myc-tag^+^/YFP^+^ THP-1 cells are thereafter referred to as *α-PD-L1 THP-1* cells (Fig. 1C, right). To evaluate circuit activation, the α-PD-L1 THP-1 cells were co-cultured with cell lines expressing different PD-L1 levels. SKOV-3 ^PD−L1+^ ovarian cancer cells and CHO-K1 ^PD−L1+^ cells exhibiting high and medium PD-L1 levels, respectively, were used as test conditions whereas SKOV-3 cells in which PD-L1 was genetically ablated (SKOV-3 ^KO^) were used as a negative control [20] (Fig. 1D). For co-colture experiments, target cells were seeded one day prior to addition of the α-PD-L1 THP-1 cells and maintained in co-colture for 48 h. We observed significant upregulation of the BFP reporter expression when α-PD-L1 THP-1 cells were co-coltured with CHO-K1 ^PD−L1+^ and SKOV-3 ^PD−L1+^ cells, whereas no activation was detected in the SKOV-3^KO^ condition (Fig. 1E-F). Notably, circuit activation correlated with the PD-L1 expression levels on the target cells, demonstrating the sensitivity of the α-PD-L1 SNIPR receptor as a sensor of PD-L1.

Beside cancer cells, PD-L1 can also be expressed by macrophages themselves, particularly, when exposed to IFN-γ [21, 22]. We measured PD-L1 expression levels in THP-1 derived macrophages by flow cytometry and confirmed that M1-like cells, polarized with IFN-γ, exhibited the highest levels of PD-L1 compared to the other polarization states (Fig. S1C).

While in previous synNotch-based studies *cis*-inhibition was the main reported consequence of ligand and receptor expression on the same cells [15], this effect was not explicitly investigated for SNIPR receptors. Therefore, we tested whether the α-PD-L1 SNIPR receptor activation was influenced by macrophage polarization state by differentiating the α-PD-L1 THP-1 cells and polarizing them to uncommitted M0, M1-like or M2-like states while in co-culture with SKOV-3 ^KO^ and SKOV-3 ^PD−L1+^ cells (Fig. S1D). Compared to monocyte-like cells, α-PD-L1 THP-1-derived macrophages showed higher basal BFP expression in absence of any target cell, potentially caused by changes associated to the acquisition of the adherent phenotype (no sender, Fig. S1D and Fig. 1E). Of note, the M1-like α-PD-L1 macrophages presented the lowest ON/OFF ratio, possibly due to a *cis*-inhibition effect from PD-L1 aboundantly expressed on these cells (Fig. S1C, D). Nevertheless, co-culture with SKOV-3 ^PD−L1+^ cells could still induce a significant increse of the fraction of BFP+ cells in all polarization states (Fig. S1D).

SynNotch-based receptor mechanism of activation tipically require cell-cell contact between the cell expressing the target and the one expressing the receptor, but PD-L1 could be released from plasma membrane with different mechanisms such as shedding, alternative splicing, inclusion in extracelluar vescicles [23, 24]. We wondered whether SKOV-3 ^PD−L1+^ cells induced activation of the α-PD-L1 receptor by releasing PD-L1 in the medium. To test this, α-PD-L1 THP-1 cells were either incubated for 48 h with conditioned medium from SKOV-3 ^KO^ and SKOV-3 ^PD−L1+^ cells (monocyte-like, Fig. S1E) or matured to macrophage-like cells and co-cultured with SKOV-3 ^KO^ in standard RPMI medium or with SKOV-3 ^PD−L1+^ conditioned medium (macrophage-like, Fig. S1E). While a small increase of the fraction of BFP+ cells could be found in both experimental conditions, the contribution of secreted forms of PD-L1 to the activation of the α-PD-L1 receptor by SKOV-3 ^PD−L1+^ cells was negligibe.

Finally, to confirm specificity of circuit activation to PD-L1 binding, we tested the activation of an anti-CD19 SNIPR, which shared the same receptor scaffold. Anti-CD19 THP-1 cells were generated as described above, PMA-treated and polarized to M0, M1-like and M2-like states, during co-culture with SKOV-3 ^KO^ or SKOV-3 ^PD−L1+^ cells, that lack the CD19 antigen [25, 26] (Fig. S1F). The extremely low BFP expression detected in all the anti-CD19 THP-1– derived macrophages in both co-culture conditions excludes unspecific activation from the SKOV-3 ^PD−L1+^ cells (Fig. S1F).

### The α-PD-L1 SNIPR receptor impairs PD-L1/PD-1 interaction

Engagement of PD-L1 by the α-PD-L1 SNIPR was initially designed to function as a sensing event that triggers transcriptional activation of downstream outputs. However, because the engineered receptor directly binds PD-L1 at the cell surface, we asked whether α-PD-L1 SNIPR engagement might also inferfere with PD-L1 interactions to its natural receptor, PD-1, through competitive or steric effects at the ligand interface. This would have important implications as PD-L1 binding to PD-1 expressed by T cells is a key mechanism driving suppression of T cell cytotoxicity and induction of T cell exhaustion [13, 20, 27].

We tested this hypothesis via a reductionist approach using a PD-1/PD-L1 blockade bioassay in which opposing effects of TCR activation and PD-1-mediated inhibitory signaling are quantitatively converted in the expression of a luciferase reporter (Fig. 1G). In this assay, disruption of PD-1/PD-L1 interaction results in enhanced TCR signaling and increased luminescence.

Unmodified wild type THP-1 cells (wt) or engineered α-PD-L1 THP-1 cells were added to co-cultures of CHO-K1 ^PD−L1+^ antigen-presenting cells (APCs) and luciferase-expressing Jurkat T cells, at THP-1:APC ^PD−L1+^ ratios of 1:1 or 3:1. An anti-PDL1 nanobody was included separately as a positive control [28], to restore TCR signaling and increase luciferase activity (Fig. 1H). As compared to control conditions, addition of α-PD-L1 THP-1 cells resulted in a significant two-fold increase in luminescence, indicating partial restoration of TCR signaling. In contrast, addition of equal number of wild-type THP-1 cells did not significantly alter reporter activity (Fig. 1H).

Collectively, a dual action of the α-PD-L1 SNIPR emerges from these results: ligand recognition initiates transcriptional circuit activation while simultaneously exerting a measurable, albeit partial, inhibitory effect on endogenous PD-1/PD-L1 interactions. This behavior highlights how synthetic receptors can intrinsically couple signal detection with local perturbation of native signaling interfaces.

### Validation of a CD47-blocker as effector output to enhance phagocytosis of CD47^+^ cancer cells

Following validation of PD-L1 sensing by the α-PD-L1 SNIPR, we next sought to establish whether ligand-dependent circuit activation could be coupled to execution of a functional output. As a prototypical effector module, we selected CV1-Fc, an affinity-enhanced SIRPα–Fc fusion protein previously shown to interfere with CD47–SIRPα interactions [29, 30].

**Fig. 2:**
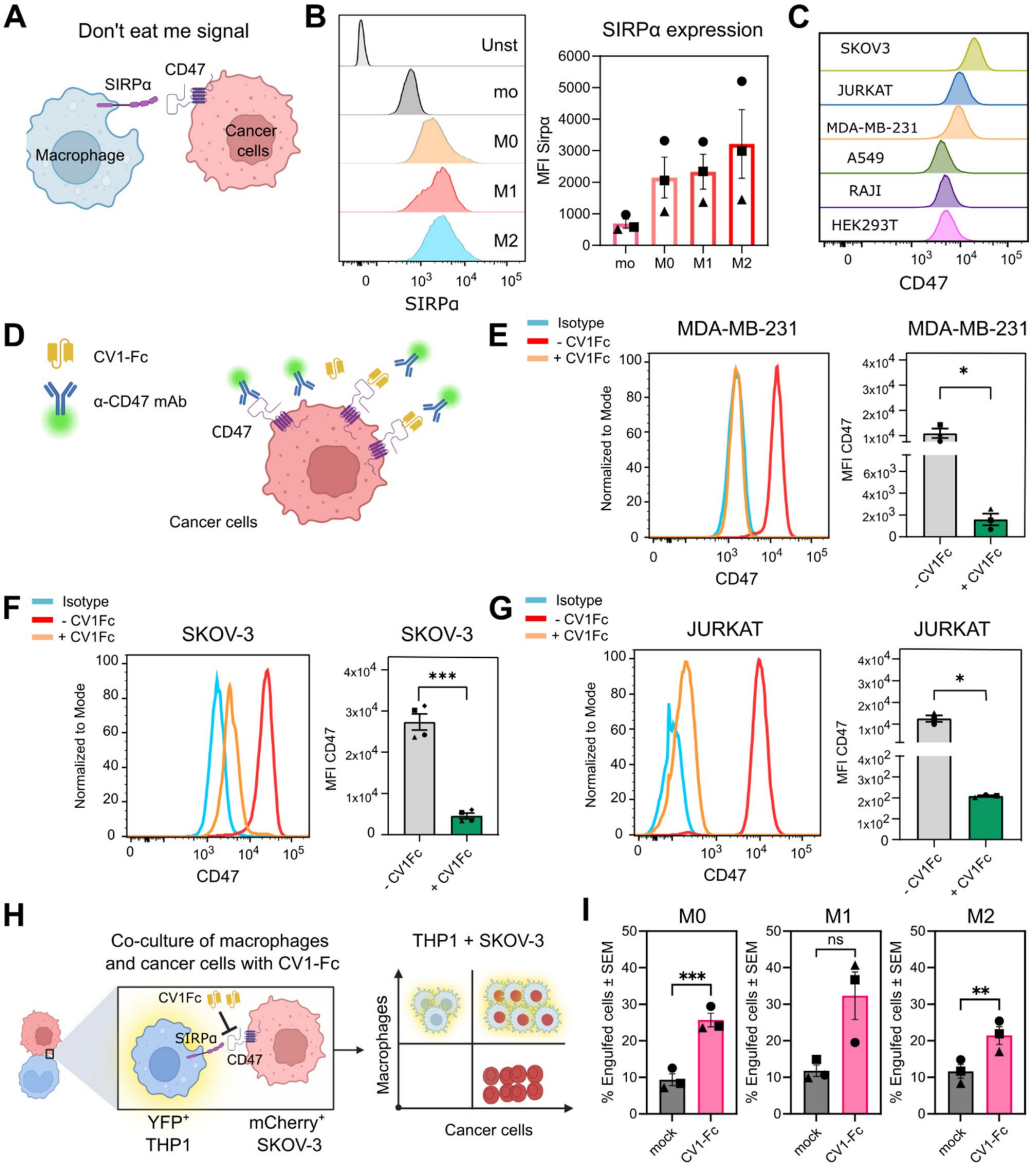
Validation of CV1-Fc as effector output to increase engulfment of CD47^+^ cancer cells. (**A**) Schematic representation of the CD47 “don’t eat me” signal. CD47 expressed on cancer cells engages signal regulatory protein α (SIRPα) on macrophages, thereby inhibiting phagocytosis. (**B**) Representative histograms of SIRPα expression in THP-1 cells measured by flow cytometry (left). Bar plots indicate SIRPα median fluorescence intensity (MFI) for each condition *n* = 3 (left). (**C**) Flow cytometry analysis of CD47 expression across a panel of human cell lines: HEK293T (human embryonic kidney), Raji (human B-cell lymphoma), A549 (human lung carcinoma), MDA-MB-231 (human breast cancer), Jurkat (human T lymphocyte), and SKOV-3 (human ovarian adenocarcinoma). (**D**) Schematic of the CD47 competition binding assay. MDA-MB-231, SKOV-3, and Jurkat cells were incubated with CV1-Fc–enriched conditioned medium prior to staining with a fluorescently labeled anti-CD47 antibody. Binding of CV1-Fc to CD47 is expected to reduce antibody binding due to competitive, steric interference. (**E-G**) Representative flow cytometry histograms and bar plots showing CD47 staining of MDA-MB-231, SKOV-3, and Jurkat cells following treatment with CV1-Fc–conditioned medium (orange) or antibody-only control (red). Reduced antibody-associated fluorescence in the presence of CV1-Fc indicates effective competition for CD47 binding. *n* = 3 (**H**) Phagocytosis assay using THP-1–derived macrophages stably expressing YFP and SKOV-3 cells stably expressing mCherry. Macrophages were polarized for 48 h and subsequently co-cultured with SKOV-3 cells for 4 h in the presence or absence of CV1-Fc–containing conditioned medium. Double-positive YFP^+^/mCherry^+^ events represent macrophages that have engulfed cancer cells. (**I**) Quantification of cancer cell engulfment by uncommitted (M0), pro-inflammatory (M1-like), and anti-inflammatory (M2-like) macrophages. Data are presented as mean ± SEM. Statistical significance was determined using paired Student’s *t* test. *n* = 3. (ns *p* > 0.5, **p < 0.01, ***p < 0.001)

CD47 is a ubiquitously expressed transmembrane glycoprotein, that functions as a “marker of self”, preventing macrophage phagocytosis through interaction with signal regulatory protein α (SIRPα) expressed on myeloid cells [31]. Because CD47 is frequently upregulated on cancer cells, it provides a dominant “don’t eat me” signal that limits macrophage-mediated tumor clearance and has therefore emerged as an important target for immunotherapy (Fig. 2A) [32, 33].

We quantified SIRPα expression in monocyte-like cells (mo), uncommitted (M0), pro-inflammatory (M1-like) or anti-inflammatory (M2-like) THP-1-derived macrophages to confirm the suitability of this system for investigating CD47–SIRPα signaling (Fig. 2B). SIRPα expression increased upon PMA treatment in all the polarization states, supporting the use of this model to study CD47-SIRPα interactions in vitro (Fig. 2B). In parallel, we assessed CD47 surface expression by flow cytometry across a panel of tumor and non-tumor cell lines, including SKOV-3, Jurkat, MDA-MB-231, A549, Raji and HEK293T cells. Among these, SKOV-3 cells exhibited the highest CD47 expression levels, providing a suitable target population for downstream assays (Fig. 2C).

To assess whether CV1-Fc could competitively block CD47 on tumor cells, we placed the CV1-Fc coding sequence with the IL-2 secretion peptide, under the GAL4 inducible promoter (UAS), and co-transfected HEK293T with a constitutively expressed GAL4-VP64 plasmid. 48 h post-transfection, the supernatant containing the CV1-Fc protein was collected (Figure S2A) and applied to MDA-MB-231, SKOV-3, and Jurkat cancer cell lines expressing CD47. Cells were subsequently stained with a fluorescently labelled anti-CD47 antibody, previously reported to compete with SIRPα for the binding to CD47 (Fig. 2D) [34, 35]. Incubation with CV1-Fc–containing conditioned medium resulted in a significant reduction of anti-CD47–associated fluorescence across all tested cancer cell lines, indicating effective competition for CD47 binding between CV1-Fc and the anti-CD47 antibody (Fig. 2E-G).

We next investigated whether CD47 neutralization and Fc receptor engagement by the CV1-Fc protein enhanced cancer cell phagocytosis, by co-culturing SKOV-3 cells with M0, M1-like or M2-like THP-1–derived macrophages. To quantify phagocytosis by flow cytometry, SKOV-3 cells were engineered to express a LifeAct–mCherry reporter, while THP-1 cells constitutively expressed a YFP reporter. 48 h post-polarization, SKOV-3 cells were seeded onto polarized THP-1–derived macrophages with conditioned medium containing the CV1-Fc protein or derived from a mock transfection (Figure S2A).

Cancer cell engulfment was quantified after 4 h of co-culture by measuring the proportion of double-positive (YFP^+^/mCherry^+^) cells (Fig. 2H). CV1-Fc conditioned medium increased the fraction of SKOV-3 cells engulfed by THP-1-derived macrophages (Fig. 2I). A separate co-culture of monocyte-like THP-1 cells with mCherry^+^ SKOV-3 cells further confirmed that the increase of mCherry+ events within the THP-1 population was due to cancer cell engulfment, while the contribution of non-specific mCherry uptake was minimal (Fig. S2B).

These results demonstrate that CV1-Fc is a valid candidate effector module for inducible execution of a phagocytic program by engineered macrophages in different polarization states.

### α-PD-L1 SNIPR–driven CD47 blockade enhances macrophage phagocytosis in vitro

**Fig. 3:**
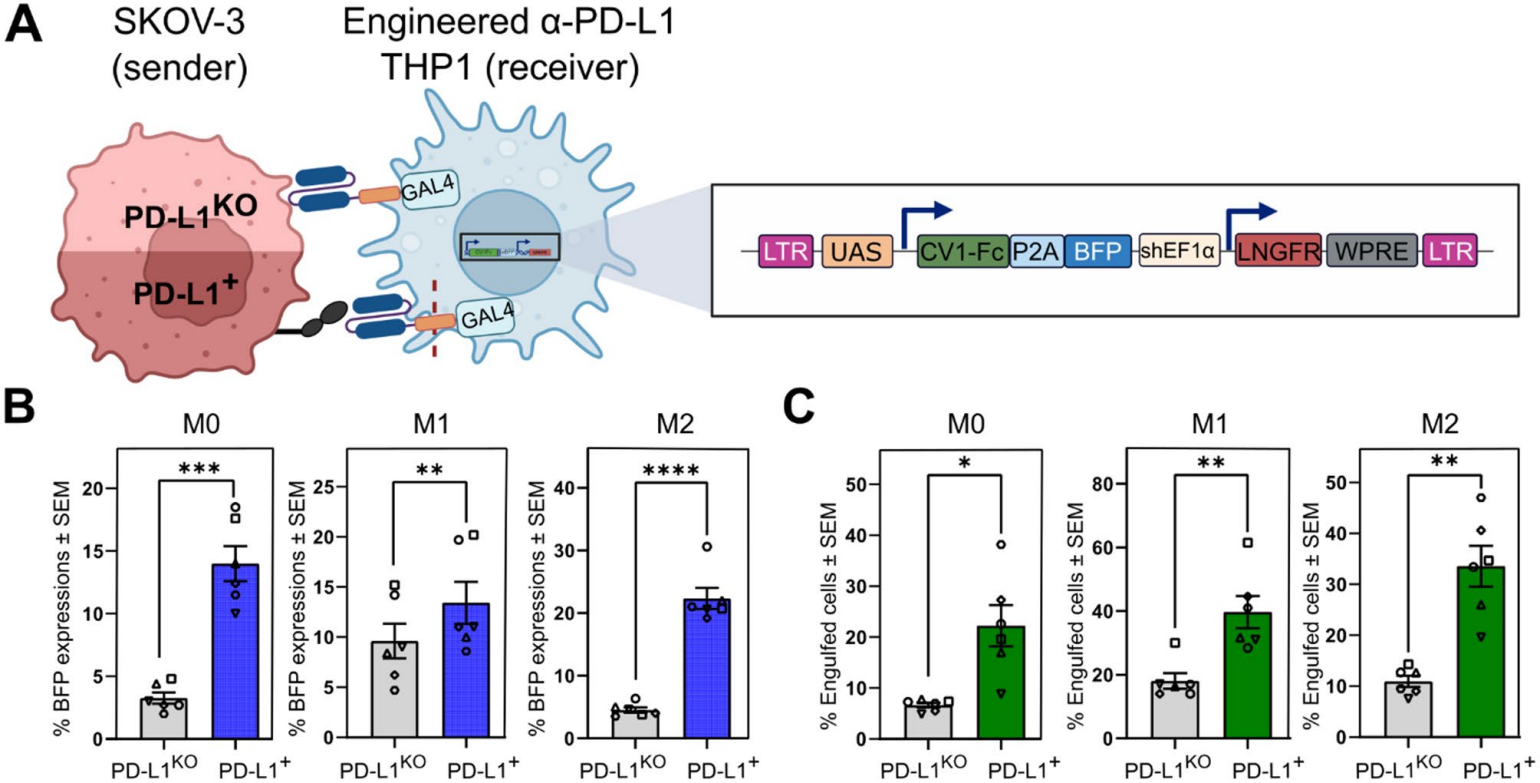
Functional validation of α-PD-L1 SNIPR circuit for CV1-Fc-mediated phagocytosis by engineered THP-1 cells. (**A**) Schematic representation of the sensor–actuator circuit. In the presence of PD-L1–deficient SKOV-3 cells (SKOV-3 ^KO^), the α-PD-L1 SNIPR remains inactive, and CV1-Fc expression is not induced. In contrast, SKOV-3 cells expressing high levels of PD-L1 (SKOV-3 ^PDL1+^) trigger SNIPR activation and downstream CV1-Fc expression. (**B-C**) Phagocytosis assays were performed by co-culturing THP-1–derived macrophages with CFSE-FITC–labeled SKOV-3^KO^ or SKOV-3^PDL1+^ cells for 48 h. Flow cytometry was used to simultaneously quantify circuit activation and macrophage-mediated phagocytosis. (**B**) BFP expression levels across uncommitted (M0), pro-inflammatory (M1-like), and anti-inflammatory (M2-like) THP-1–derived macrophages. Cells exposed to PD-L1–expressing targets exhibited increased BFP expression (blue bars) compared with ligand-negative conditions (gray bars), confirming ligand-dependent circuit activation. (**C**) Fraction of THP-1 derived macrophages that engulfed cancer cells identified by gating the CD11b^+^ THP-1 cells and, within this population, the FITC^+^ events. Exposure to PD-L1–expressing cancer cells resulted in significantly higher phagocytic activity (green bars) compared with PD-L1–negative conditions (gray bars). Statistical significance was determined using paired Student’s *t* test. *n* = 6 (*p < 0.05, **p < 0.01, ***p < 0.001, ****p < 0.0001)

To establish a closed-loop architecture linking ligand detection to effector execution, we next integrated the CV1-Fc module into the α-PD-L1 SNIPR circuit to enable its conditional expression upon PD-L1 sensing. For this purpose, we generated a lentiviral construct containing two transcriptional units (TUs). The first TU comprises the GAL4-responsive promoter (UAS) driving the expression of CV1-Fc, followed by a P2A self-cleaving peptide and a BFP reporter. The second TU encodes a constitutively expressed LNGFR cell-surface marker to enable selection of transduced cells (Fig. 3A). THP-1 cells were co-transduced with the α-PD-L1 SNIPR sensor and the responsive actuator construct. Double positive Myc-tag^+^/LNGFR^+^ cells were isolated by FACS to obtain a population where both sensor and actuators were integrated (Fig. S2C).

THP-1 cells engineered with the sensor-actuator circuit, thereafter referred to as *α-PD-L1 CV1-Fc THP-1*, were differentiated and uncommitted M0, M1-like and M2-like macrophages were co-cultured with SKOV-3 ^KO^ or SKOV-3 ^PDL1+^ cells. Cancer cells were labelled with CFSE–FITC before seeding onto THP-1-derived macrophages. After 48 h, cells were harvested and analyzed by flow cytometry using BFP expression as a readout of circuit activation. The macrophage sub-population was identified by gating the CD11b^+^ cells and, within these cells, the FITC^+^ events marked macrophages that had engulfed cancer cells (Fig. S2D). Co-culture with SKOV-3 ^PDL1+^ cells induced a significant increase of BFP expression in all differentiated macrophages compared to SKOV-3^KO^ cells (Fig. 3B and Fig. S3A). Notably, the M1-like cells showed the lowest relative increase of BFP^+^ cells, similar to what observed with the α-PD-L1 THP-1 cells driving only BFP reporter expression (Fig. 3B, S3A and Fig. S1D). Quantification of the FITC^+^ events confirmed that PD-L1-dependent activation of the α-PD-L1 SNIPR resulted in significantly enhanced engulfment of cancer cells by THP-1-derived macrophages in all polarization states (Fig. 3C and Fig. S3B).

Although receptor activation results in BFP expression and the secretion of the CV1-Fc protein in the extracellular environment, we wondered whether the cells marked by BFP were more likely to engulf cancer cells. Gating the FITC^+^ events within the BFP^+^ and BFP^-^ populations showed that, indeed, the former exhibited higher cancer cell engulfment across all polarization states in both SKOV-3^KO^ and SKOV-3 ^PDL1+^ co-culture conditions (Fig. S3C). These results suggest that the secretion of the CV1-Fc protein may have both autocrine and paracrine action, resulting in higher engulfment capacity of the producing cells.

Moreover, while the α-PD-L1 CV1-Fc THP-1 cells showed PD-L1 dependent increase of engulfment, non-engineered THP1 cells displayed comparable levels of phagocytosis toward SKOV-3 ^PDL1+^ and SKOV-3 ^KO^ cells, indicating that enhanced engulfment was dependent on circuit activation (Fig. S3D).

Collectively, these results demonstrate that macrophages engineered with a sensor–actuator circuit can translate ligand-specific recognition via the α-PD-L1 receptor into a functional phagocytic output across distinct polarization states. This behaviour underscores the capacity of sensor–actuator circuits to implement closed-loop control of innate immune cell functions in a context-dependent manner.

### Transcriptional control of α-PD-L1 SNIPR expression by macrophages regulatory elements

**Fig. 4:**
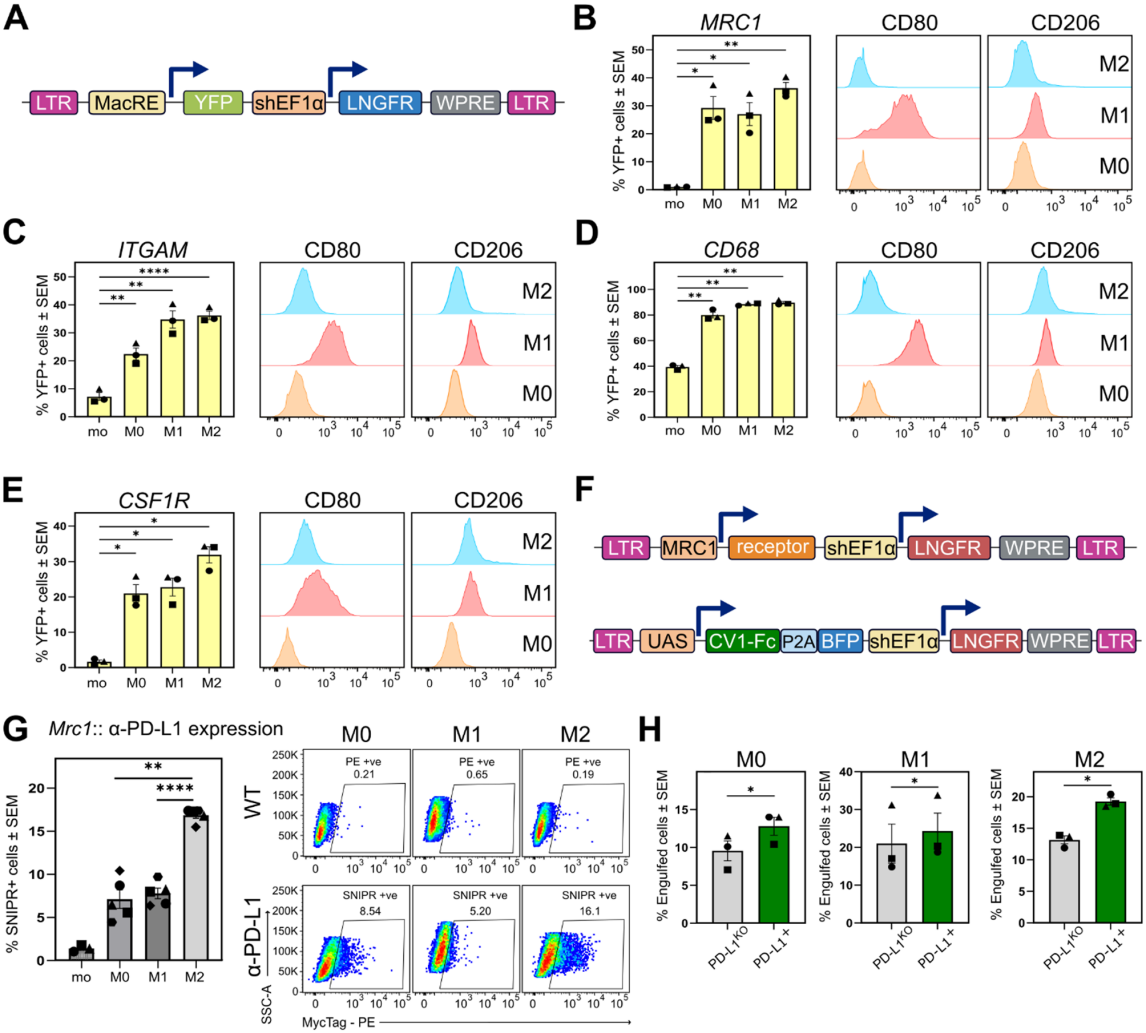
Characterization of macrophage regulatory elements (MacRE) and transcriptional control of α-PD-L1 SNIPR expression. (**A**) Schematic of the macrophage regulatory element (MacRE) reporter system. (**B-E**) Flow cytometry analysis of MacRE activity following 48 h of macrophage polarization. Representative histograms of the expression of phenotypic markers for M1-like (CD80) and M2-like (CD206) states are shown together with YFP expression driven by the *MRC1* (**B**), *ITGAM* (**C**), *CD68* (**D**), and *CSF1R* (**E**) promoter-derived fragments. Bar plots indicate the mean ± SEM of the fraction of YFP^+^ cells across all conditions. Statistical significance was determined using repeated-measures one-way ANOVA with Dunnett’s multiple comparisons test (* adjusted *p* < 0.05; ** adjusted *p* < 0.01; *** adjusted *p* < 0.001; **** adjusted *p* < 0.001), *n* = 3. (**F**) Schematic of the two lentiviral vectors embedding the MRC1-driven α-PD-L1 SNIPR and inducible CV1-Fc actuator constructs. (**G**) Flow cytometry analysis of α-PD-L1 SNIPR expression driven by the *MRC1* promoter in monocytic-like cells (mo) and following differentiation and polarization. Fraction of cells expressing the α-PD-L1 SNIPR receptor across polarization states (left) and representative pseudocolor plots upon staining with an anti-Myc Tag antibody of unmodified (WT) and engineered (α-PD-L1) THP-1 cells (right). Bar plots indicate mean ± SEM. Statistical significance was determined using paired Student’s *t* test. *n = 3–5* (**p < 0.01, ****p < 0.0001). (**H**) Flow cytometry analysis to quantify THP-1 that engulfed cancer cells after phagocytosis assay performed by co-culturing polarized THP-1-derived macrophages and SKOV-3^KO^ or SKOV-3^PDL1+^ cells for 48 h. Data are presented as mean ± SEM. Statistical significance was determined using Student’s *t* test. *n* = 3 (*p < 0.05, **p < 0.01)

While constitutive expression of the α-PD-L1 SNIPR enables its deployment across diverse cell types, it may also lead to unintended circuit activation due to stochastic encounters with PD-L1-expressing cells. To limit unwanted induction, we leveraged the ability of synthetic or endogenous-derived promoters to confer context-dependent control of gene expression [36–39]. We sought to restrict expression of the synthetic receptor to committed macrophages by leveraging endogenous macrophage regulatory elements (MacREs) derived from *MRC1*, *CD68*, *CSF1R* and *ITGAM* promoters [40–43]. Promoter fragments encompassing these MacREs were cloned upstream of a YFP fluorescent reporter in lentiviral vectors, followed by a constitutively expressed LNGFR marker to identify the transduced cells (Fig. 4A). Each MacRE construct was independently integrated into THP-1 cells and, after sorting the LNGFR^+^ cells, YFP expression was quantified by flow cytometry prior to macrophages differentiation (mo) and following polarization into uncommitted M0, pro-inflammatory (M1-like) and anti-inflammatory (M2-like) macrophages (Fig. 4B-E and S4).

YFP expression was minimal at the monocytic-like state (mo) across all MacREs, whereas differentiation into macrophages increased the proportion of YFP^+^ cells (Fig. 4B-E, S4B). The CD68-derived promoter exhibited detectable activity already in monocytes and was further activated across all polarization states (Fig. 4D). In contrast, *MRC1-*, *CSF1R-* and *ITGAM*-derived promoters showed little to no activity in monocytes and were activated at different levels by macrophage differentiation and polarization (Fig. 4B-E). Among these, the *MRC1*-derived promoter, exhibited the lowest basal activity at the monocyte-like state (mo) with a high proportion of YFP^+^ cells following polarization, with preferential activation in the M2-like phenotype. These results establish that MacREs can function as endogenous transcriptional filters, enabling restriction of synthetic gene expression in macrophages. To assess whether this regulatory layer could be integrated into the α-PD-L1 SNIPR circuit, we placed the receptor coding sequence under the control of the MRC1-derived promoter.

The construct was integrated into THP-1 cells previously transduced with the inducible CV1-Fc actuator module, without additional selection for cells bearing the complete circuit (Fig. 4F). Engineered THP-1 cells were then differentiated and polarized, and α-PD-L1 SNIPR expression was quantified by anti-myc staining. Consistent with the YFP reporter data, receptor expression was barely detectable in the monocytic-like state and increased upon macrophage maturation and polarization, particularly in M2-like macrophages (Fig. 4G).

We next evaluated whether *MRC1-*driven receptor expression translated into functional circuit output. Engineered THP-1 were differentiated, polarized and co-cultured with SKOV-3^PD−L1+^ or SKOV-3^KO^ cells. While increased phagocytosis was observed across all polarization states, the M1-like cells showed the lowest relative induction, indicating that MacRE-based transcriptional control can limit circuit activity in defined macrophage phenotypes (Fig. 4H).

Together, these findings demonstrate that endogenous regulatory elements can be integrated as a second control layer within a synthetic receptor circuit, enabling hierarchical regulation in which extracellular sensing and cellular state jointly determine system output. This layered architecture provides a general strategy for improving specificity and robustness of synthetic circuits operating in heterogeneous cellular environments.

## Discussion

In this study, we implemented a synthetic receptor–based circuit that couples detection of a defined extracellular ligand to execution of a programmable intracellular response in monocytic-like cells. Rather than treating immune checkpoints exclusively as therapeutic targets, we conceptualized PD-L1 as an information-bearing signal that can be repurposed as an input for context-dependent output expression. By leveraging a synthetic Notch-based receptor, ligand engagement is converted into transcriptional activation of user-defined outputs, thereby decoupling signal sensing from endogenous PD-L1 signaling pathways.

To test this novel circuit, we opted for a human monocytic THP-1 cell line as a reductionist in vitro system that reproduces several key properties of human macrophages following differentiation, including phagocytosis, polarization-dependent gene expression, and sensitivity to immune-regulatory signals [44, 45].

Our results demonstrate that PD-L1 sensing by the α-PD-L1 SNIPR correlates with ligand abundance and can partially interfere with PD-1/PD-L1–mediated inhibitory signaling in a reductionist assay. Importantly, this interference arises as a consequence of receptor engagement rather than as an explicit design goal, underscoring how synthetic receptors can simultaneously function as signal transducers and competitive binders within complex signaling networks. While the magnitude of checkpoint interference is limited relative to soluble antagonists, this behaviour highlights an intrinsic coupling between sensing and modulation of target functions that may be relevant when designing future multi-functional receptors.

Beyond signal detection, we demonstrate that ligand-responsive activation can be linked to execution of a phagocytic program through inducible expression of a CD47-blocking effector. Here, CV1-Fc was employed as a model output to illustrate how synthetic circuits can deploy context-dependent effectors in response to defined extracellular cues, rather than as an optimized solution for macrophage-mediated clearance [29, 30]. Conditional CV1-Fc expression enhanced engulfment of PD-L1-expressing cancer cells across multiple macrophage polarization states.

However, our results highlight also differences among the phenotype tested that have important implications for future circuit design. Pro-inflammatory cells exhibited the highest absolute engulfment but the lowest relative difference of BFP expression, whereas anti-inflammatory cells showed the highest PD-L1 dependent relative engulfment. We hypothesize that the high PD-L1 levels reached in M1-like cells might be responsible for *cis*-inhibition effect, reducing activation of the circuit in this phenotype.

BFP expression is a proxy for production of the CV1-Fc protein, which by simultaneously blocking the “don’t eat me signal” CD47 and binding the Fc receptors is able to induce cancer cell engulfment. However, the overall engulfment also depends on the expression of others “eat me” and “don’t eat me” signals, inhibiting receptors and Fc receptors that could be differentially expressed across polarization states. In this perspective, pro-inflammatory environment resulted in the lowest ON/OFF ratio of the circuit but the overall highest fraction of engulfed cells.

A central challenge in synthetic circuit design is balancing responsiveness with specificity, particularly in systems exposed to heterogeneous or fluctuating inputs. To address this, we introduced a second regulatory layer by constraining receptor expression through endogenous macrophage regulatory elements. The *MRC1*-derived promoter reduced receptor expression in monocytic like cells, while also reducing circuit output in the pro-inflammatory phenotypes. Although the overall magnitude of phagocytosis appeared decreased compared to constitutive receptor expression, direct comparisons are limited by the absence of cell sorting for complete integration in the *MRC1*-driven α-PD-L1 SNIPR circuit. Nevertheless, this multi-layer regulation illustrates how endogenous transcriptional programs can be harnessed to refine synthetic control in complex cellular systems.

Some limitations of the present study should be acknowledged. All experiments were conducted in vitro using a human monocytic cell line, and circuit behaviour was evaluated under controlled co-culture conditions. Future studies will be required to validate the robustness of this architecture in primary macrophages or in more heterogeneous environments. In addition, the present work does not attempt to optimize effector potency or address system performance under dynamic signalling regimes, which will be important considerations for future extensions of this framework.

Despite these limitations, this study establishes a modular control architecture that integrates sensing, transcriptional decision-making, and effector deployment within an innate immune chassis. Further, by framing immune-regulatory ligands as programmable inputs rather than fixed biological endpoints, our work contributes a systems-level perspective on how synthetic circuits can be used to interrogate and rewire immune regulation.

Together, this work advances a synthetic systems biology approach to immune cell programming, in which layered regulatory architectures enable controlled interpretation of complex extracellular information and execution of defined cellular responses.

## Materials and methods

### Cell culture

HEK293T, MDA-MB-231, A549, THP-1, Jurkat, and Raji cell lines used in this study were purchased from the American Type Culture Collection (ATCC). The SKOV-3^KO^ and SKOV-3 ^PD-L1+^ ovarian cancer cell lines were kindly provided by the Guedan laboratory [20]. CHO-K1 ^PD-L1+^ APC and luciferase-expressing Jurkat effector cells were used as part of the PD-1/PD-L1 blockade bioassay from Promega following manufacturing istructions.

HEK293T, MDA-MB-231, A549, and SKOV-3 cells were maintained in Dulbecco’s modified Eagle medium (DMEM; Gibco) supplemented with 10% fetal bovine serum (FBS, non–USA origin; Sigma-Aldrich), 1% penicillin/streptomycin (Sigma-Aldrich), 1% L-glutamine (Sigma-Aldrich), and 1% MEM non-essential amino acids (Sigma-Aldrich). THP-1, Jurkat, and Raji cells were cultured in RPMI-1640 medium supplemented with 10% heat-inactivated FBS, 1% penicillin/streptomycin, 1% L-glutamine, and 1% MEM non-essential amino acids. All cell lines were maintained at 37 °C in a humidified incubator with 5% CO₂.

### Cell transfection for CV1-Fc fusion protein production

HEK293T cells were transfected using PEI^®^ transfection reagent (Polysciences) according to the manufacturer’s instructions, at a DNA (µg) to PEI (µL) ratio of 1:3. To generate conditioned medium containing the CV1-Fc fusion protein for competition and phagocytosis assays, 5 × 10⁶ HEK293T cells were seeded in T75 flasks in a final volume of 14 mL of complete medium. Cells were transfected with 10 µg of total plasmid DNA. Mock transfection control was generated by transfecting a CMV:: BFP expression vector. Conditioned medium was collected 48 h later, clarified by centrifugation, aliquoted, and stored at − 80 °C until use.

### Lentiviral plasmid design and cloning

The sequence encoding the α–PD-L1 SNIPR was assembled downstream of a PGK promoter. From N- to C-terminus, the construct comprises a CD8 signal peptide to ensure membrane localization; a Myc epitope tag to enable detection of surface expression of the SNIPR using a PE-conjugated anti-Myc antibody (Cell-Signaling Technology #3739); an anti–PD-L1 nanobody [18]; the core regulatory domain of the SNIPR receptor, consisting of the human Notch1 transmembrane domain, and juxtamembrane regions (FMYVAAAAFVLLFFVGCGVLLS-RKRRR) and a GAL4-VP64 transcriptional activator [17].

The inducible CV1-Fc actuator construct was generated starting from a plasmid kindly shared by the Ogris laboratory [30]. The coding sequence assembled downstream of UAS responsive elements included an IL-2 signal peptide, the high-affinity SIRPα fragment, CV1, fused to the Fc region of human IgG1, followed by a P2A self-cleaving peptide and a BFP reporter. To enable identification of cells harboring the complete inducible construct, a second transcriptional unit (TU) driven by a short EF1α promoter (shEF1α) and encoding a truncated low-affinity nerve growth factor receptor (ΔLNGFR) was included downstream of the actuator TU. Expression of ΔLNGFR was detected using an anti–human CD271 (NGFR) antibody (BioLegend, #345132).

Macrophage regulatory element (MacRE) reporter constructs were generated by cloning regulatory regions derived from *MRC1*, *ITGAM*, *CD68*, or *CSF1R* upstream of a YFP reporter gene [40–43]. Similar to the actuator construct, a second cassette consisting of the shEF1α promoter and ΔLNGFR was included to mark transduced cells. The MRC1:: α-PD-L1 SNIPR construct was generated by placing the α–PD-L1 SNIPR coding sequence under the control of the *MRC1*-derived promoter.

All lentiviral constructs were cloned using In-Fusion cloning (Takara Bio, #638951) into a modified pHR’SIN: CSW lentiviral backbone kindly provided by the Roybal group [17]. The anti-CD19 SNIPR receptor and the GAL4 reporter construct, containing UAS responsive elements driving BFP expression and a PGK promoter driving constitutive YFP expression, were also kindly provided by the Roybal group [17].

### Lentivirus production

5 × 10^6^ HEK293T cells were seeded from 16 to 18 h before transfection in T75 flask in a total volume of 15mL of complete DMEM. The following day, cells were transfected with a plasmid mixture containing the pHR transfer vector, the pMD2.G envelope plasmid (Addgene #12259), and the psPAX2 packaging plasmid (Addgene #12260) at a ratio of 2:1:1. FuGENE^®^ HD transfection reagent (Promega, #E2311) was used at a DNA (µg) to FuGENE (µL) ratio of 1:3. Within 24 h from transfection, the culture medium was replaced to remove residual DNA and transfection reagent. Lentiviral supernatants were collected at 48 and 72 h post-transfection, filtered through a 0.45-µm filter and concentrated using Lenti Concentrator (OriGene Technologies #TR30026) according to the manufacturer’s instructions. Concentrated virus was resuspended in cold DPBS, aliquoted and stored at − 80 °C until use.

### Cell transduction and lentiviral titer determination

THP-1 cells were engineered using a reverse transduction protocol, in which complete medium, lentiviral supernatant, cells, and polybrene (10 µg/mL; Sigma-Aldrich) were added sequentially in this order. Transductions were typically performed in 24- or 12-well plates using 2.5–3.0 × 10⁵ cells per well in a total volume of 750 µL or 1 mL, respectively. Cells were transduced either simultaneously or sequentially, depending on the experimental design. 48 h post-transduction, the culture medium was replaced to remove residual lentiviral particles and polybrene. Between 72 and 96 h post-transduction, expression of circuit components was assessed by flow cytometry through quantification of YFP reporter expression for the integration of the GAL4 reporter cassette, or by staining with PE-conjugated anti-Myc antibody (Cell Signaling Technology, #3739) and Pacific Blue™-conjugated anti–CD271 (NGFR) antibody (BioLegend, #345132) to confirm the integration of sensor and actuator modules.

Lentiviral titers were determined directly in THP-1 cells seeded. Lentiviral supernatants were added to cells at dilution ratios of 1:300, 1:600, and 1:900 relative to the total culture volume. The fraction of transduced cells for each circuit module was quantified by flow cytometry as described above. Viral titers were calculated using samples yielding 20–40% transduction efficiency, according to the following formula:

$$\:\frac{T\:=N\:*\:F\:*\:D}{VT}$$

where T = Titer (TU/mL); N = Number of cells used for Titration; F = Fraction of cells successfully transduced; D = Dilution Factor; VT = Transduction Volume, mL.

### THP-1 differentiation into macrophages and polarization

THP-1 differentiation and polarization were performed using different plate formats according to experimental requirements adapting previously published protocols [19]. 4.2 × 10⁵ or 2.5 × 10⁵ THP-1 cells were seeded, respectively, into 12- or 24-well tissue culture–treated plates in complete RPMI-1640 medium supplemented with phorbol 12-myristate-13-acetate (PMA; Sigma-Aldrich, #J63916.LB0) at a final concentration of 10 nM. Cells were incubated for 48 h to induce differentiation before refreshing the medium to remove PMA. THP-1–derived macrophages were then kept in complete RPMI medium without any cytokine to obtain uncommitted cells (M0) or polarized toward a pro-inflammatory state (M1-like) by supplementing the medium with 10 ng/mL lipopolysaccharide (LPS; Thermo Fisher Scientific, #00-4976-93) and 20 ng/mL interferon-γ (IFN-γ; Bio-Techne, #285-IF-100) or toward an anti-inflammatory state (M2-like) by adding 20 ng/mL interleukin-4 (IL-4; PeproTech, #200-04-20UG) and 20 ng/mL interleukin-13 (IL-13; PeproTech, #200-13-10UG). Cells were incubated with polarization stimuli for 48 h. Flow cytometric analysis was performed immediately thereafter to evaluate the expression of canonical M1 marker using Super Bright™ 702-conjugated anti-CD80 (B7-1) monoclonal antibody (BioLegend, #67-0809-42) and M2 marker with BV421-conjugated anti-CD206 antibody (BioLegend, #564062). SIRPα expression in THP-1-derived macrophages was measured with a PE-conjugated anti-SIRPα (CD172a) antibody (BioLegend, #372104), whereas PD-L1 expression was detected using a PE-conjugated anti-PDL1 (CD274, B7-H1) antibody (Thermo Fisher Scientific, #12-5983-42).

### In vitro characterization of α-PD-L1 SNIPR activation

To quantify circuit activation in monocyte-like cells, SKOV-3 and CHO-K1 cells were seeded one day prior to co-culture to allow adherence. Engineered THP-1 cells were subsequently added on top of the adherent cells. Different co-culture ratios were used to achieve comparable densities of adherent target cells before addition of THP-1 cells in suspension. α-PD-L1 THP-1 cells were seeded at a ratio of 2.5:1 relative to the initial number of SKOV-3 ^KO^ or SKOV-3 ^PD-L1+^ cells and at a ratio of 1.25:1 relative to CHO-K1 ^PD-L1+^ cells (Promega). After 48 h of co-culture, cells in suspension were collected, washed with DPBS, and resuspended in FACS buffer. The fraction of cells expressing the BFP reporter within the YFP^+^ population was quantified by flow cytometry to assess circuit activation.

To quantify activation of the circuit in anti-PDL1 and anti-CD19 THP-1-derived macrophages, 5 × 10⁵ engineered cells were seeded in 6-well plates and differentiated with PMA as described above. After 48 h, SKOV-3 ^KO^ and SKOV-3 ^PDL1+^ cells were added at a macrophage: cancer cell ratio of 1:2 together with cytokines to induce polarization. Co-cultures were incubated at 37 °C for an additional 48 h. Following incubation, cells were prepared for flow cytometry analysis as described below.

To test contact dependence of the activation of the anti-PDL1 SNIPR receptor, conditioned medium from a confluent flask of SKOV-3 ^PDL1+^ cells was harvested and spun down to remove cells in suspension. Co-cultures of uncommitted THP-1-derived macrophages with SKOV-3 ^KO^ cells in either standard RPMI or diluting the conditioned medium with equal volume of RPMI were analysed after 48 h to compare BFP expression. Alternatively, conditioned media from SKOV-3 ^KO^ and SKOV-3 ^PDL1+^ cells were added to monocyte-like α-PDL1 THP-1 cells and BFP expression was measured 48 h later.

### PD-1/PD-L1 blockade bioassay

The capacity of the α-PD-L1 SNIPR to interfere with PD-1/PD-L1 interaction was tested by adding engineered THP-1 cells to the PD-1/PD-L1 Blockade bioassay (Promega). 3.3 × 10⁴ CHO-K1 ^PD-L1+^ antigen-presenting cells (APCs) were seeded 24 h prior to co-culture. The following day, the same number of PD-1^+^ Jurkat effector cells were added to the CHO-K1 cultures together with either α-PD-L1 SNIPR–expressing THP-1 cells or wild-type THP-1 cells at THP-1: CHO-K1 ratios of 1:1 or 3:1. As a positive control for PD-1/PD-L1 pathway inhibition, an anti–PD-L1 nanobody [28] was added to the co-cultures of CHO-K1 APCs and PD-1 effector cells at a final concentration of 1 µg/mL. Luminescence was measured after 6 h of co-culture using a GloMax^®^ Discover system (Promega).

### CD47 competition assay

7.0 × 10⁴ MDA-MB-231 and 4.0 × 10⁴ SKOV-3 cells were seeded one day prior to the competition assay in 24-well plates, in a final volume of 0.5 mL of complete medium. 4.0 × 10⁵ Jurkat cells were seeded in 24-well plates on the same day of the competition assay. Cells were incubated for 1 h at 37 °C with 0.5 mL of conditioned medium, harvested from HEK293T cells transfected to secrete CV1-Fc. Cells not incubated with CV1-Fc–containing conditioned medium were used as untreated controls. Following incubation with or without CV1-Fc, cancer cells were washed and stained with a FITC-conjugated anti-CD47 antibody (BioLegend, #11-0479-41) or a FITC-conjugated mouse IgG1 κ isotype control. Fluorescence associated with antibody binding was quantified by flow cytometry to assess competition for CD47 binding.

Staining with the same FITC-conjugated anti-CD47 antibody was also used to determine CD47 expression across different cancer cell lines such as A549, Jurkat, SKOV-3, MDA-MB-231and Raji.

### Phagocytosis assay

To assess the effect of CV1-Fc conditioned medium, 4.0 × 10⁵ THP-1 cells constitutively expressing YFP were seeded in 12-well plates in complete RPMI medium adding PMA to induce maturation to macrophages. After 48 h, polarization stimuli were added as described above. Following additional 48 h, SKOV-3 cells constitutively expressing an mCherry reporter were prepared at a macrophage: cancer cell ratio of 1:3 and resuspended in conditioned medium produced by HEK293T cells expressing CV1-Fc or a mock transfection control (CMV:: BFP). Cancer cells were subsequently added to the macrophage cultures and incubated at 37 °C for 4 h, after which cells were harvested and analyzed by flow cytometry. Passive uptake of mCherry by THP-1 cells was tested by co-culturing undifferentiated monocyte-like YFP^+^ THP-1 cells with mCherry^-^ or mCherry^+^ SKOV-3 cells overnight. mCherry signal detected within the two YFP^+^ THP-1 populations was then compared by flow cytometry.

To assess the effect of the α-PD-L1/CV1-Fc circuit, 4.8 × 10⁵ engineered THP-1 cells were seeded in 6-well plates and differentiated with PMA as described above. After 48 h, SKOV-3 ^KO^ and SKOV-3 ^PDL1+^ cells were prepared at a macrophage: cancer cell ratio of 1:2 and labeled with FITC-CellTrace™ CFSE Cell Proliferation Kit (Thermo Fisher Scientific, #C34554) according to the manufacturer’s instructions. Labeled tumor cells were then added to the macrophage cultures together with cytokines to induce polarization. Co-cultures were incubated at 37 °C for additional 48 h. Following incubation, cells were prepared for flow cytometry analysis as described below. Macrophages were stained with an PE-Cy5–conjugated anti–CD11b antibody (BioLegend, #301308), and FITC-CFSE fluorescence within the CD11b^+^ population was quantified to assess cancer cell engulfment.

### Flow cytometry and cell sorting

All cells were analyzed with a BD CELESTA™ cell analyzer (BD Biosciences). THP-1-derived macrophages were detached using sterile-filtered Accutase^®^ solution (Sigma-Aldrich, #A6964-100ML). After 10 min of incubation, residual cells still attached were lifted with cell scrapers (Biosigma #010153). After detachment, cells were resuspended in 1 mL of complete medium, washed once with DPBS, and incubated with Fc Receptor Binding Inhibitor (Invitrogen, #14-9161-73) for 15 min at room temperature. Antibody staining was performed in a final volume of 50 µL of BD Horizon™ Brilliant Stain Buffer (BD Biosciences #563794) for 20 min at room temperature, washed once with DPBS, and resuspended in FACS buffer (DPBS +2mM EDTA + 1%FBS). Population of live cells and single cells were selected according to FSC/SSC parameters. Data analysis was performed with FlowJo 10.10.0 Software (BD Life Sciences).

For cell sorting, all cells were analyzed with BD FACSMelody™ Cell Sorter. THP-1 cells were prepared in sterile conditions as described above for general flow cytometry analysis. To select the cells in which both sensor and actuators were integrated antibody staining was performed with PE-conjugated anti-Myc antibody (Cell Signaling Technology #3739) and Pacific Blue™ anti–human CD271 (NGFR) antibody (BioLegend #345132). Before sorting, cells were resuspended in 3mL of FACS medium and passed into polypropylene tubes through a 70 μm cell strainer (BD Biosciences #340605). Cells were collected in complete RPMI medium, spun down, resuspended in fresh medium and seeded in multi-well plates at an approximate concentration of 5 × 10^5^ cells/ml.

### Statistical analysis and figure preparation

Bar plots represent the mean of three to six independent experiments. Error bars indicate the standard error of the mean (SEM), and symbols of different shapes represent individual experimental values. Unless explicitly specified, statistical significance was determined using: two-sided paired Student’s t test for comparison between one control condition and one or two test conditions (*p < 0.05; **p < 0.01); repeated measures one-way ANOVA with Dunnett’s or Tukey’s multiple comparisons test for comparisons, respectively, among one or multiple control conditions and multiple test conditions (* adjusted p value < 0.05; ** adjusted p value < 0.01). Statistical analysis was performed using either R version 4.3.1 or GraphPad Prism 10.6.1.

For figure generation, R packages including tidyverse, rstatix, ggplot2, ggpubr, ggsignif, dplyr or GraphPad Prism were used to generate data plots and Biorender was used to generate all the illustrations. Inkscape v1.4 was used to assemble figure panels.

## Supplementary Information

Below is the link to the electronic supplementary material.

Supplementary Material 1

## Data Availability

All data generated or analysed during this study are included in this published article and its supplementary information files.
