# Supplementary material for: Engineering a PD-L1–sensing synthetic receptor for programmable macrophage response
Source: J Biol Eng. 2026 Jun 6;20:123. doi: 10.1186/s13036-026-00708-y (PMC13459757; doi:10.1186/s13036-026-00708-y)
Supplement: Supplementary file 1 [file 13036_2026_708_MOESM1_ESM.docx]

**Supplementary Fig.1 Validation of α-PD-L1 SNIPR activation in THP-1-derived macrophages.**

**(A)** Schematic of the differentiation and polarization protocol. THP-1 monocytes were differentiated into macrophage-like cells by 48 hours of treatment with PMA and subsequently polarized toward a pro-inflammatory M1-like phenotype (IFN-γ and LPS) or an anti-inflammatory M2-like phenotype (IL-4 and IL-13). Flow cytometry analysis was performed 48-hours post-polarization to assess surface expression of CD80 (M1 marker) and CD206 (M2 marker). **(B)** CD80 and CD206 expression measured by flow cytometry (left). Representative histograms of CD80 and CD206 (right). Bar plots representing mean ± SEM of CD80 median fluorescence intensity (MFI) and the percentage of CD206-positive cells for each condition. Statistical significance was determined by using repeated-measures one-way ANOVA with Dunnett’s multiple comparisons test (* adjusted p<0.05, ** adjusted p<0.01, *** adjusted p<0.001). *n=3*. **(C)** Representative histograms of PD-L1 expression in monocytic-like THP-1 (mo) and THP-1-derived macrophages. **(D)** Activation of the α-PD-L1 SNIPR receptor in THP-1-derived macrophages. BFP expression in M0, M1-like or M2-like THP-1–derived macrophages alone (no sender, dark grey bars), or upon 48 hours of co-culture with SKOV-3^KO^ (magenta bars) or SKOV-3^PDL1+^ (cyan bars) cells. Bar plots represent mean ± SEM. Statistical significance was determined using a two-way ANOVA followed by Tukey’s test. *n=3* (^ns^ adjusted p>0.05, * adjusted p<0.05). **(E)** Activation of the α-PD-L1 SNIPR receptor by PD-L1 not bound to the plasma membrane was tested incubating undifferentiated α-PD-L1 THP-1 cells with conditioned medium from SKOV-3^KO^ or SKOV-3^PDL1+^ cells for 48 hours (monocyte-like) or co-culturing uncommitted α-PD-L1 THP-1-derived macrophage with SKOV-3^KO^ in standard RPMI medium or with conditioned medium from SKOV-3^PDL1+^ cells for 48 hours (macrophage-like). Statistical significance was determined using Student’s *t* test. *n=3* (^ns^ p>0.05; *p<0.05). **(F)** Unspecific SNIPR receptor activation was tested by co-culturing for 48 hours α-CD19 THP-1-derived macrophages with SKOV-3^KO^ (dark grey bars) or SKOV-3^PDL1+^ (magenta bars) cells. Data are presented as mean ± SEM. Statistical significance was tested using a two-way ANOVA followed by Šidák's multiple comparisons test. *n=3* (^ns^ adjusted p>0.05).

**Supplementary Fig.2 Characterization of the CV1-Fc actuator module as secreted protein or downstream of the α-PD-L1 receptor**

**(A)** Production of CV1-Fc conditioned medium by transfection of HEK293T cells. A BFP reporter was included downstream of the CV1-Fc coding sequence separated by a P2A self-cleaving peptide as proxy for the production of the CV1-Fc protein. Transfection of a plasmid bearing a CMV::BFP cassette was used as mock control. Blue fluorescence was detected by EVOS inverted fluorescence microscopy. **(B)** To test passive uptake of mCherry by the THP-1 cells, monocyte-like THP-1 cells were co-cultured overnight with mCherry^+^ or mCherry^-^ SKOV-3 cells. Flow cytometry pseudocolor plots show the fraction of mCherry^+^ cells gated in the YFP^+^ population. **(C)** Schematic of the two lentiviral vectors used to generate the α-PD-L1 CV1-Fc THP-1 cells, bearing the sensor–actuator synthetic circuit. The lentiviral vector encoding CV1-Fc comprises two tandem transcriptional units (TUs). The first TU contains the GAL4-responsive UAS promoter upstream of the CV1-Fc coding sequence followed by a P2A sequence and the BFP reporter, enabling fluorescence-based detection of circuit activation. In the second TU, the constitutive shEF1α promoter drives the expression of truncated ΔLNGFR as a transduction marker. Following co-transduction, Myc⁺/ΔLNGFR⁺ cells were sorted to purify cells that integrated both sensor and actuator. **(D)** Representative contour plots showing the gating strategy to quantify circuit activation (BFP+ cells) and cancer cell engulfment (CFSE-FITC+ cells).

**Supplementary Fig.3 α-PD-L1 CV-1 Fc circuit activation and cancer cells engulfment by THP-1-derived macrophages.**

(**A-C**) Representative contour plots originating from uncommitted (M0), pro- inflammatory (M1-like), and anti-inflammatory (M2-like) α-PD-L1 CV-1 Fc THP-1-derived macrophages cultured alone (no sender) or co-cultured with SKOV-3^KO^ or SKOV-3^PDL1+^ cells for 48 hours. BFP expression (**A**) was used as a proxy of circuit activation, whereas FITC+ events (**B**) represented the fraction of engulfed CFSE-FITC SKOV-3 cells. **(C)** Starting from the same samples shown in A and B, the contour plots indicate the fraction of FITC^+^ events within the BFP^+^ and BFP^-^ populations of THP-1-derived macrophages in co-culture with SKOV-3^KO^ or SKOV-3^PDL1+^ cells. **(D)** SKOV-3^KO^ (grey bars) or SKOV-3^PDL1+^ (green bars) cell engulfment by wild-type THP-1-derived macrophages. Bar plots show mean ± SEM of three independent experiments. Statistical significance was determined using a paired Student’s *t* test. (^ns^ p>0.05).

**Supplementary Fig.4 Design and validation of the MacREs reporter system.**

**(A)** Representative pseudocolor plots of the lentiviral transduction of THP-1 cells with the MacRE:: YFP constructs derived from *ITGAM*, *MRC1*, *CD68*, or *CSF1R* promoters. The fraction of THP-1 cells expressing the constitutive ΔLNGFR transduction marker and YFP driven by the MacRE, are shown together with untransduced cells (THP-1 wt). **(B)** Representative pseudocolor plots showing the fraction of THP-1 cells expressing the YFP reporter downstream of each MacRE in monocytic-like THP-1 (mo) and THP-1-derived macrophages compared with untransduced cells (WT).
